# A new option to prevent fistulas in anterior urethroplasty in patients with kippered urethra: the tunica vaginalis flap

**DOI:** 10.1590/S1677-5538.IBJU.2020.1058

**Published:** 2021-04-20

**Authors:** Luciano A. Favorito, Fernando Salles da Silva, José Anacleto de Resende

**Affiliations:** 1 Hospital Federal da Lagoa Rio de JaneiroRJ Brasil Hospital Federal da Lagoa, Rio de Janeiro, RJ, Brasil; 2 Universidade do Estado do Rio de Janeiro Unidade de Pesquisa Urogenital Rio de JaneiroRJ Brasil Unidade de Pesquisa Urogenital, Universidade do Estado do Rio de Janeiro, UERJ, Rio de Janeiro, RJ, Brasil; 3 Universidade do Estado do Rio de Janeiro Departamento de Urologia Rio de JaneiroRJ Brasil Departamento de Urologia, Universidade do Estado do Rio de Janeiro, UERJ, Rio de Janeiro, RJ, Brasil

**Keywords:** Urethra, Fistula, Tunica Intima

## Abstract

The objective of this study is describing a technique with the use of a tunica vaginalis flap (TVF) to cover the suture line during anterior urethroplasty in patients with kippered urethra due to chronic indwelling catheterization (CIC). We studied 5 patients (mean age=50.2) with a neurogenic bladder that developed urethral erosion after a long period of CIC. Foley catheter was removed on the 14th postoperative day. One patient developed wound infection and utethrocutaneous fistula, which was conservatively managed and after 12 months of follow-up all the patients didn't report difficulties in intermittent self-catheterization. In conclusion, a urethroplasty with TVF technique may be a viable method for repairing penile urethral erosions, but further studies are required with a bigger sample to confirm our results.

## INTRODUCTION

The use of flaps is very important to protect the suture line and avoid fistulas in surgical corrections of penile urethral strictures. The tunica vaginalis flap (TVF) was used as an additional cover of suture line and fistula prevention in hypospadias and epispadias with an acceptable complication rate and good cosmetic results (1). The use of TVF as the dorsal component of a two-stage urethroplasty in anterior urethral strictures presented significant fibrosis and this kind of flap is not suitable in Bracka surgery (2).

Urethral strictures occur in about 5 to 20% of patients as a complication of chronic indwelling catheterization (CIC) (3). Penile urethral erosion (kippered urethra) is a rare complication of CIC, with some studies reporting it to occur more frequently in men with neurogenic bladder (3). There are techniques described for repairing the ventral urethral erosions but a standardized approach is not yet available (4, 5).

TVF was used in anterior urethral strictures corrections (6) but studies about surgical techniques for repairing the ventral erosions in patients with CIC are scarce in literature. Recently we published a video with the use of TVF to prevent fistulae in a patient with kippered urethra (7). The objective of this paper is to describe a simple surgical technique to prevent urethral fistulae in patients with urethral erosions using a tunica vaginalis flap.

## SURGICAL TECHNIQUE

**Figure 1:**
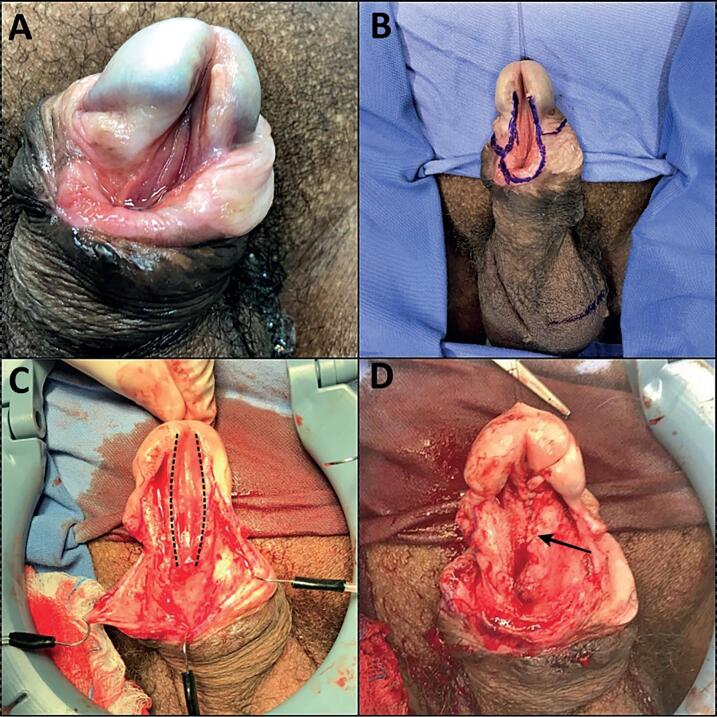
The figure shows the initial step of the surgical procedure using the the tunica vaginalis flap (TVF) in anterior urethroplasty for a patient with urethral erosion after chronic indwelling catheterization (CIC): A) Preoperative aspect of the urethral erosion by CIC in a 46 years-old patient; B) Demarcation of the subcoronal incision and around urethral erosion; C) Dissection and separation of the the urethral plate (dashed line) from the penile skin and dartos; D) Urethral tubularization in a 2 planes continuous suture of its margins with 5-0 PDS (arrow).

This study was carried out in accordance with the ethical standards of the hospital's institutional committee on human experimentation. We prospectively analyzed patients admitted to our facility with diagnosis of kippered urethra (Figure-1A) between January 2018 and February 2020.

In the operating room (OR), a single dose of cefazolin (2g) was given as a systemic prophylactic antibiotic against Gram-positive and Gram-negative bacteria. The external genitalia were shaved to remove hair from the surgical site.

**Figure 2:**
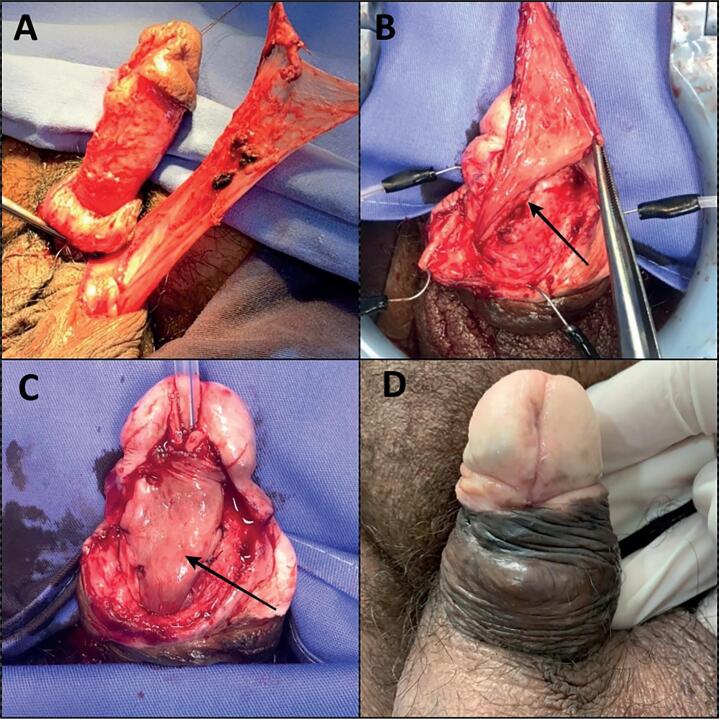
The figure shows the second step of the surgical procedure using tunica vaginalis flap (TVF) in anterior urethroplasty for a patient with urethral erosion after chronic indwelling catheterization (CIC): A) Confection of a vascularized tunica vaginalis flap (TVF) from the left testis; B) The figure shows the confection of a submucosal tunnel with TVF (arrowhead) transposition; C) Final aspect of the coverage of the urethral suture line with the TVF (arrowhead) and D) The postoperative aspect one month after the urethral catheter removal.

The patients were placed supine, disinfected and draped sterilely. The surgical incision was delimited with a marking pen (Figure-1B) and the urethral plate was separated from the penile skin and dartos tissue by an incision at its limits with the adjacent tissue following dissection (Figure-1C). After mobilization of the urethral margins, urethral tubularization was performed in a 2-plane continuous suture of its margins with 4-0 PDS (Figure-1D). Luminal diameter was calibrated with a 16Fr Foley catheter. The next step was the access of the testicle by a subcutaneous tunnel and confection of a 5 to 6cm vascularized TVF (Figure-2A). This tissue was used to cover the urethral suture (Figure-2B and Figure-2C) and after the TVF fixation we reconstructed the glans and closed the penile skin. Patients were discharged on the 2nd postoperative day, and a Foley catheter was maintained for 14 days. The mean follow-up time was 12.25 months (range: 10-14 months). Uroflowmetry was not performed because the patients had no spontaneous urination. The final aspect 4 weeks after the catheter removal in one of the cases is demonstrated in Figure-2D.

## RESULTS

We studied 5 patients with neurogenic bladder who developed urethral erosion after a long period of CIC (Table-1). The patient's ages ranged from 20 to 69 years (mean age=50.2). The mean urethral defect length was 4.92cm ± (range: 3.4 to 5.8cm). The 5 patients had urethral erosions and difficulties in maintaining CIC.

**Table 1:** The table shows demographic data of the 5 patients studied. We can observe the patients' age (in years), the length of the urethral erosion (in centimeters), the comorbidities and the etiology that led to the use of a urethral catheter.

Patient	Age (years)	Length of urethral erosion (cm)	Etiology	Comorbidities
1	20	3.4	Neurogenic bladder	Down syndrome
2	67	5.5	Neurogenic bladder	BPH, diabetes mellitus
3	69	4.5	Neurogenic bladder	BPH, diabetes mellitus
4	47	5.4	Neurogenic bladder	Spinal cord injury
5	48	5.8	Neurogenic bladder	Spinal cord injury
Mean	50.2	4.92		

**BPH** = Benign prostatic hyperplasia.

Only 1 patient (20%) developed, after the surgery wound infection and urethra-cutaneous fistula, which was conservatively managed with the use of 2g of cephalexin for 10 days and with urethral catheterization for 14 days. The other 4 patients did not report difficulties in CIC after at least 10 months of follow-up. The procedure had no impact on sexual function, and the final aspect had no additional changes except for the scar, even in the patient with wound infection.

## DISCUSSION

The use of indwelling urinary catheters could be associated with urethral erosion involving portions or complete erosion of the glans and penile shaft and in these cases the urethral reconstruction is necessary to restore the penile anatomy (8, 9). In our sample we observed only one immediate complication after the catheter removal in a patient that developed a wound infection and a small urethra-cutaneous fistula, which was conservatively managed with antibiotics and urethral catheterization. We believe that the worst result in this patient may be due to other conditions (neurogenic bladder, diabetes mellitus and had both legs amputated with difficulties in personal hygiene). There are several factors associated to urethra-cutaneous fistula after urethroplasty and the main ones are the etiology of stricture, stricture length, urinary infection, cutaneous infection and multiple previous treatments (10). The other 4 patients in our sample did not report difficulties in CIC after at least 10 months of follow-up. The procedure had no impact in sexual function, and the final aspect had no additional changes except for the scar, even in the patient with wound infection.

The results of urethral reconstruction in patients with spinal cord injuries are poor, probably because of local issues as impaired wound healing and limited tissue reserves, also, we believe that the superposition of the suture lines, associated with the ventral skin and dartos fascia erosion could increase the risk of fistula formation. Thus, the lack of a well vascularized tissue covering the urethral suture is a concern in these patients (11).

TVF is useful for hypospadia correction (1) and we believe that the same results will be obtained with the use of this flap in urethral erosions. As far as we know, there are no reports about the use of this technique in cases of urethral erosion after CIC. This technique is easy to perform and in our initial cases we had good results in 80% of them, with minor complications in only one case, which was resolved with the use of a bladder catheter.

This study has important limitations that must be mentioned: single center study with small sample size and short follow-up, which makes the evaluation of long-term complications, such as urethral diverticulum, impossible.

Therefore, this initial study suggests that the use of a TVF may be a viable method to cover the urethral suture during reconstruction in patients with urethral erosions. Further studies with a larger number of patients carried out in several centers with long-term follow-up are required to validate the effectiveness of this technique.
